# Reliability and Validity of the Function Impairment Screening Tool in Chinese Older Adults

**DOI:** 10.3389/fmed.2021.720607

**Published:** 2021-10-13

**Authors:** Yaxin Zhang, Pan Liu, Yiming Pan, Ying Li, Li Zhang, Yun Li, Lina Ma

**Affiliations:** Department of Geriatrics, China National Clinical Research Center for Geriatric Medicine, Xuanwu Hospital, Capital Medical University, Beijing, China

**Keywords:** functional impairment, reliability, validity, screen, older adults

## Abstract

**Background:** Physical function gradually decreases with age in older adults, affecting their independence and quality of life and leaving them prone to adverse outcomes. Despite the importance of assessing function for older adults, most studies have focused on disability and paid less attention to functional impairment. Thus, given the lack of valid and practical methods for evaluating functional impairment for older adults, we developed the function impairment screening tool (FIST) using the Delphi method.

**Objective:** This study aimed to evaluate the reliability and validity of the FIST in Chinese older adults.

**Methods:** A total of 489 participants aged 60 years or older, and who had completed the FIST were included. A subgroup of 50 participants completed the FIST a second time, 1 week after the first round, and the test–retest reliability was evaluated using the intraclass correlation coefficient (ICC). Reliability was tested using Cronbach's alpha. Validity was examined using exploratory factor analysis. Criterion-related validity was assessed using correlations between the FIST and the Barthel Index activities of daily living (ADL), Lawton, and Brody instrumental activities of daily living (LB-IADL).

**Results:** The Cronbach's alpha coefficient for the FIST was 0.930 (*P* < 0.001). The test–retest reliability was good, with an ICC of 0.928 (95% confidence interval [0.874, 0.960]). Exploratory factor analyses revealed one factor accounting for 60.14% of the scale's variance and the load values of every item were >0.4 (0.489–0.872). The correlation coefficient was 0.572 (*P* < 0.001) between the FIST score and ADL, and was 0.793 (*P* < 0.001) between the FIST score and IADL. The FIST score was positively correlated with walking speed (*r* = 0.475, *P* < 0.001) and grip strength (*r* = 0.307, *P* < 0.001), and negatively correlated with age (*r* = −0.588, *P* < 0.001) and Fried frailty phenotype (*r* = −0.594, *P* < 0.001).

**Conclusion:** The FIST is a reliable and valid instrument for assessing physical function impairment in older adults.

## Introduction

Physical function gradually decreases with age in older adults, affecting their independence and quality of life, leaving older adults more vulnerable to adverse outcomes such as falls, hospitalization, and death (1–3). Declining function increases the cost of public healthcare systems and imposes the burden of additional care on families (4). In 2020, the Chinese population aged 60 or above was 264.02 million, accounting for 18.7% of the country's total population (5). Currently, China faces an enormous healthcare challenge. The World Health Organization (WHO) defined “healthy aging” as a process of maintaining functional ability to enable wellbeing in older age (6). The disease concept is increasingly being replaced by the concept of functional consequences in geriatric medicine (7). The original conceptual model on disability was proposed by Nagi. In his model, disability was defined as having problems in activities of daily life (ADL) (8). In 2001, WHO redefined disability by the International Classification of Functioning, Disability, and Health (ICF). Disability is an umbrella term, involving impairments, activity limitations, and participation restrictions (9). WHO developed Disability Assessment Schedule-3.6 for assessing disability which had very good psychometric properties (10). The frequently used tools for disability were Barthel Index (BI), Lawton and Brody instrumental activities of daily living (LB-IADL), and Katz Index (KI) (11). Physical function encompasses all body functions, activities, and participation (12). Physical function impairment is generally considered to occur earlier in the process of disability (13). Early identification and intervention are of great importance. Therefore, it is especially important to assess the functional status of older adults, as interventions for function impairment can prevent or delay the occurrence of disability and adverse events and promote healthy aging.

However, most previous studies have focused on disability (14–16), while paying less attention to the early stage of disability, that is, functional impairment. This may be attributable to the lack of valid tools to assess functional impairment in older adults. Though there were basic activities of daily living (BADL) and IADL for evaluating the performance in activities of daily living, but the BADL and IADL had low sensitivity for detecting mild function deficits. The advanced activities of daily living (AADL) can assess more complex functional performance, but some items of AADL were not suited to Chinese older adults; go to church, for example (17). We developed the function impairment screening tool (FIST) for Chinese older adults using the Delphi method (18, 19).

The FIST contains 16 items, divided into three domains: ability to perform activities of daily living, ability to engage in domestic life, and ability to engage in social activities (19). The FIST is a self-reported questionnaire, and it is low-cost and easy to use. Meanwhile, the FIST meets the definition of physical function, while avoiding the ceiling effect. However, there has been no formal psychometric evaluation of the FIST. This study aimed to evaluate the validity and reliability of the FIST in Chinese older adults.

## Methods

### Participants

Participants aged 60 years or older were recruited from the inpatient department at the Department of Geriatrics at Xuanwu Hospital, Capital Medical University. Exclusion criteria included a Mini-Mental State Exam (MMSE) score <23 (20) and a BI score <40. Based on the criteria, 500 older adults were enrolled in the study. The study was approved by the Ethics Committee of Xuanwu Hospital Capital Medical University, and was registered, number: ChiCTR1900028382. All participants agreed to be included in the study and provided their written informed consent before participation.

### Data Collection

Demographic characteristics and history of chronic diseases were collected for all participants by geriatricians or postgraduate students. All of the data gatherers were trained using standardized procedures on assessment a week before the study. ADL and IADL were assessed using the BI (21) and LB-IADL (22), respectively. Cognitive function was assessed *via* MMSE (20). We assessed frailty using the Fried frailty phenotype including weakness, slowness, exhaustion, inactivity, and weight loss (23). Weakness was assessed by measuring the dominant maximal grip strength according to standardized procedures. We used a hand dynamometer (EH101; Camry, Guangdong Province, China) to measure the grip strength. Participants sat in an upright position with the arm in adduction and the elbow flexed at 90 degrees (24). Two tests were carried out for the dominant hand, with a 1-min interval between two tests. We recorded the maximal grip strength of the two tests. Slowness was assessed by performing the 4-m walking test. A 4-m line was marked on the ground, and participants were asked to walk at their usual speed. Every participant was tested twice, and the shorter time was recorded (25). The cutoff values for grip strength and walking speed were derived from previous reports in Chinese older populations (26, 27). The total score of the Fried frailty phenotype is 5. Older adults with a score ≥ 3 were classified as “frail,” those with 0 were considered “robust,” and scores 1–2 indicated “prefrail.”

### Function Impairment Screening Tool

The FIST is a self-report questionnaire for assessing physical function in older adults. The FIST contains 16 items, divided into three domains: ability to perform activities of daily living (including eating, washing, going to toilet, dressing, moving in and out of bed, and bathing), ability to engage in domestic life (including housekeeping, handling finances, handing medications, lifting 5 kg of weight, and up and down stairs), and ability to engage in social activities (including using transportation, shopping, walking for 250 m, using telephone, and physical exercise) (19). Each item has three levels—independence, partial dependence, and complete dependence—and is scored as 1, 0.5, and 0, respectively. The total score range for all 16 questions is 0 to 16; higher score indicates better physical function (19).

### Statistical Analysis

The data were analyzed using SPSS version 19.0 (SPSS Inc., Chicago, IL, USA). The figures were drawn using GraphPad Prism version 7.0 software (GraphPad Software Inc., CA, USA). Quantitative variables were described as mean with standard deviation (±SD), and categorical variables were described as the number with percentages (%). The overall scale reliability was tested using Cronbach's alpha coefficient. Meanwhile, we also calculated item-to-total correlations (ITTC) the split-half coefficient, and the Guttman split-half coefficient. Internal Consistency Reliability and split-half testing measure reliability. The ITTC is a measure of the reliability of a multi-item scale and evaluates the correlation between an individual item and the total score without that item. An acceptable value should be >0.50 (28). Total score test–retest reliability was assessed by two-way random intraclass correlation coefficients (ICC) for absolute agreement. An indication of good reliability was values of ICC ≥ 0.7, and excellent reliability was indicated by ICC ≥ 0.9 (29). We also plotted the difference of scores between the test–retest measures using the Bland and Altman plot (30). The Bland and Altman plot is a graphical method to evaluate a bias and compare two measurements of the same variable. The construct validity was calculated by exploratory factor analysis (EFA). The EFA was run using the principal components analysis. The number of factors retained was based on the scree test (31, 32). An item was retained if its factor loading was >0.40. As the total score of FIST was non-normal distribution, the criteria validity of the FIST was measured using Spearman's correlation coefficient with the FIST and ADL, IADL scores. The absolute value of Spearman's correlation coefficient was used, for which <0.5, >0.7, and 0.5–0.7 mean weak correlation, strong correlation, and moderate correlation, respectively (33). Furthermore, we calculated the correlation between FIST score and age, physical performance, and physical frailty for exploring some related factors. All statistical tests were two-tailed, and a *P* of <0.05 was considered statistically significant.

We calculated sample size using subject-to-item ratios, allowing for a minimum of a 20:1 subject-to-item ratio (34). The minimum sample size was 320 subjects with a total of 16 items. The number of participants was greater than the number required (34, 35). We calculated the sample size of test–retest based on the COSMIN standards (36). We set an ICC value of 0.85 as a priori for the optimal target level of reliability. The minimal sample size was 35 and was calculated using two repeated measures with a confidence interval (CI) of 0.2. We evaluated the test–retest reliability with 50 older adults, which was greater than the above minimal sample size (37).

## Results

In total, 500 participants were enrolled from Jan 2020 to Mar 2021, 11 older adults declined to participate. A total of 489 older participants were included in the final analysis. The average age of participants was 74.01 (±9.60) years. Two hundred eighty-three (57.87%) of the participants were male. The mean FIST total score for all of the participants was 14.15 (±3.25). The mean ADL and IADL scores were 91.43 (±14.33) and 7.18 (±1.78), respectively. The mean values of grip strength and walking speed were 27.07 (±9.81) kg and 0.89 (±0.30) m/s, respectively. The demographic characteristics and chronic disease data of the participants are shown in Table 1.

**Table 1:** Characteristics of study participants.

**Characteristic**	**Total (*N* = 489)**
Age, years	74.01 ± 9.60
**Gender**	
Male	283 (57.87)
Female	206 (42.13)
**Marital status**	
Never married	2 (0.41)
Married	391 (79.96)
Divorced/Separated	8 (1.63)
Widowed	88 (18.00)
**Education level**	
Illiteracy	13 (2.66)
Primary school	53 (10.84)
Middle school	178 (36.40)
Senior High school	126 (25.77)
University	105 (21.47)
Post-graduate	14 (2.86)
BMI, kg/m^2^	25.37 ± 3.70
**Chronic diseases**	
Hypertension	372 (76.07)
Coronary heart disease	188 (38.45)
Diabetes mellitus	183 (37.42)
Cerebrovascular disease	108 (22.09)
Chronic kidney disease	59 (12.07)
Osteoarthropathy	141 (28.83)
SBP, mmHg	137.05 ± 18.02
DBP, mmHg	73.95 ± 11.48
FIST score	14.15 ± 3.25
IADL score	7.18 ± 1.78
ADL score	91.43 ± 14.33
MMSE score	27.64 ± 2.66
Grip strength, kg	27.07 ± 9.81
Walking speed, m/s	0.89 ± 0.30

*Continuous variables are expressed as mean ± SD and categorical variables are expressed as frequency (%)*.

*ADL, Activity of Daily Living; BMI, Body Mass Index; DBP, diastolic blood pressure; FIST, function impairment screening tool; IADL, instrumental activities of daily living; MMSE, Mini-Mental State Examination; SBP, systolic blood pressure*.

### Reliability

**Figure 1:**
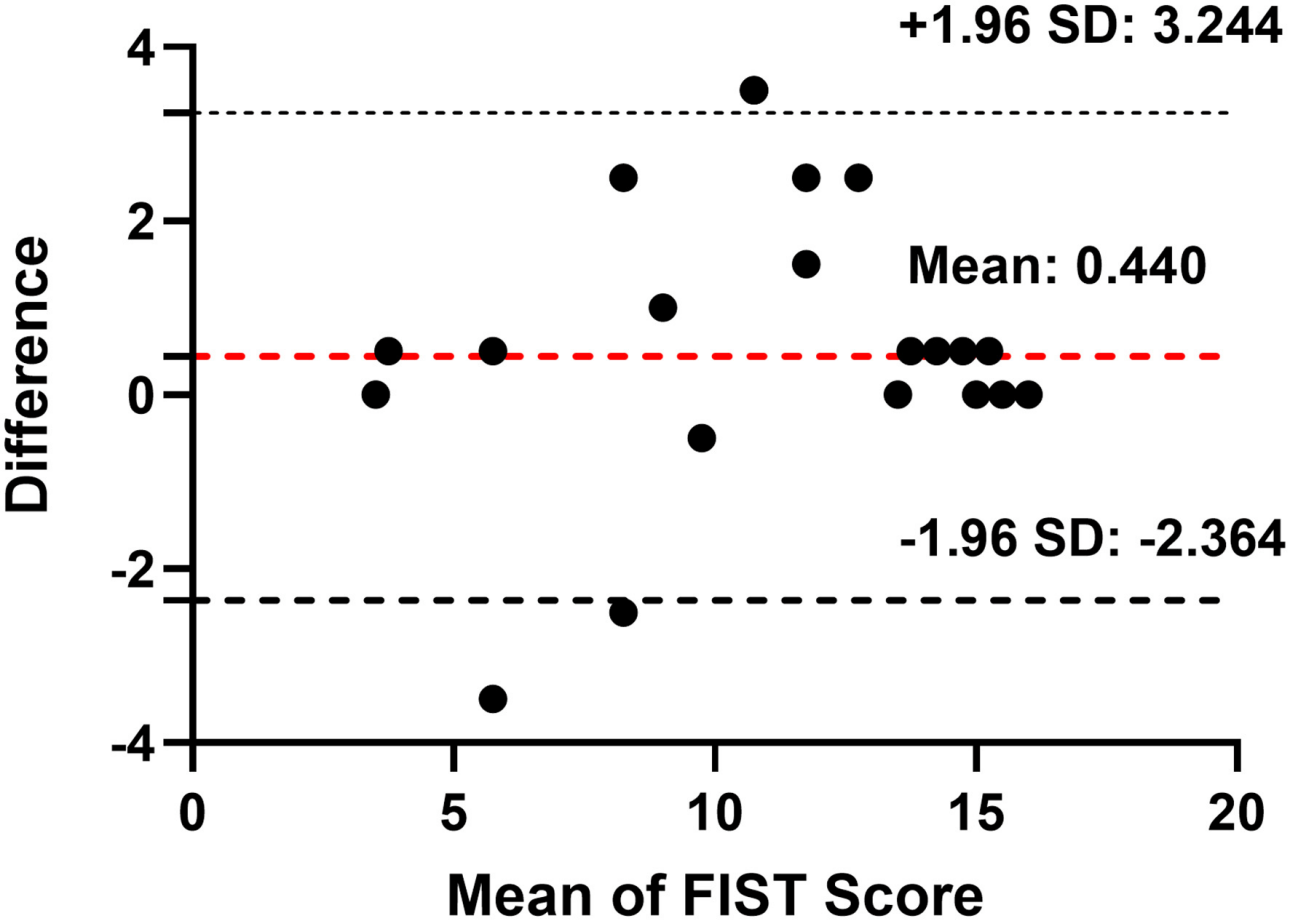
Bland-Altman plot: mean change of FIST scores between test and retest and its 95% confidence interval (CI). Red dotted lines indicate the mean change of FIST scores and black dotted lines indicate two standard deviations of the mean difference. SD, standard deviation.

We calculated the internal consistency reliability using the Cronbach coefficients. The Cronbach's alpha coefficient of the FIST total score was 0.930 (Table 2), and the split-half coefficient and Guttman split-half coefficient were 0.924 and 0.881, respectively (Table 3). A subgroup of 50 participants completed the FIST a second time after 1 week. The two-way random ICC test–retest reliability was 0.928 (*P* < 0.001; Table 4). The mean change of FIST scores and its 95% CI between test and retest were presented in the Bland–Altman plot (Figure 1).

**Table 2:** Scale overall reliability.

**Items**	**Corrected item-to-total correlation (CITC)**	**Cronbach's alpha if item deleted**
**Ability of daily living**		
Eating	0.541	0.931
Washing	0.655	0.928
Going to toilet	0.781	0.926
Dressing	0.707	0.927
Moving in and out of bed	0.778	0.926
Bathing	0.828	0.922
**Ability of domestic life**		
Housekeeping	0.751	0.923
Handling finances	0.805	0.921
Handling medications	0.745	0.923
Lifting 5 kg of weight	0.753	0.924
Up and down stairs	0.767	0.923
**Ability of social activities**		
Using transportation	0.851	0.920
Shopping	0.875	0.919
Walking for 250 m	0.463	0.943
Using telephone	0.642	0.928
Physical exercise	0.596	0.929

**Table 3:** Internal consistency reliability and split-half reliability of the FIST scale and each factor.

	**Internal consistency reliability coefficient**	**Split-half reliability coefficient**	**Guttman split-half coefficient**
Total scale	0.930	0.924	0.881
Ability of daily living	0.929	0.952	0.945
Ability of domestic life	0.892	0.779	0.745
Ability of social activities	0.781	0.771	0.634

**Table 4:** ICC coefficient for test–retest reliability of FIST total score.

	**ICC**	**95%CI**	***F*** **-test with true value 0**			
			**Value**	**df1**	**df2**	***P-*value**
Single measures	0.928	0.874–0.960	29.09	49	49	<0.001
Average measures	0.963	0.933–0.979	29.09	49	49	<0.001

*CI, confidence interval; ICC, intraclass correlation coefficient*.

### Validity

Exploratory factor analysis was used to calculate construct validity. The Kaiser–Meyer–Olkin (KMO) measure of sampling adequacy was 0.917, and the Bartlett's test of sphericity (BTS) was 7,904.477 (*P* < 0.001), which indicated that the data were suitable for factor analysis. Exploratory factor analyses revealed one factor accounting for 60.14% of the scale's variance, and the load values of every item were >0.4 (0.489–0.872; Table 5).

**Table 5:** Load values of each item in the FIST.

**Item**	**Factor 1**
Eating	0.646
Washing	0.762
Going to toilet	0.860
Dressing	0.804
Moving in and out of bed	0.864
Bathing	0.872
Housekeeping	0.750
Handling finances	0.804
Handling medications	0.805
Lifting 5 kg of weight	0.811
Up and down stairs	0.771
Using transportation	0.846
Shopping	0.865
Walking for 250 m	0.489
Using telephone	0.729
Physical exercise	0.625

**Figure 2:**
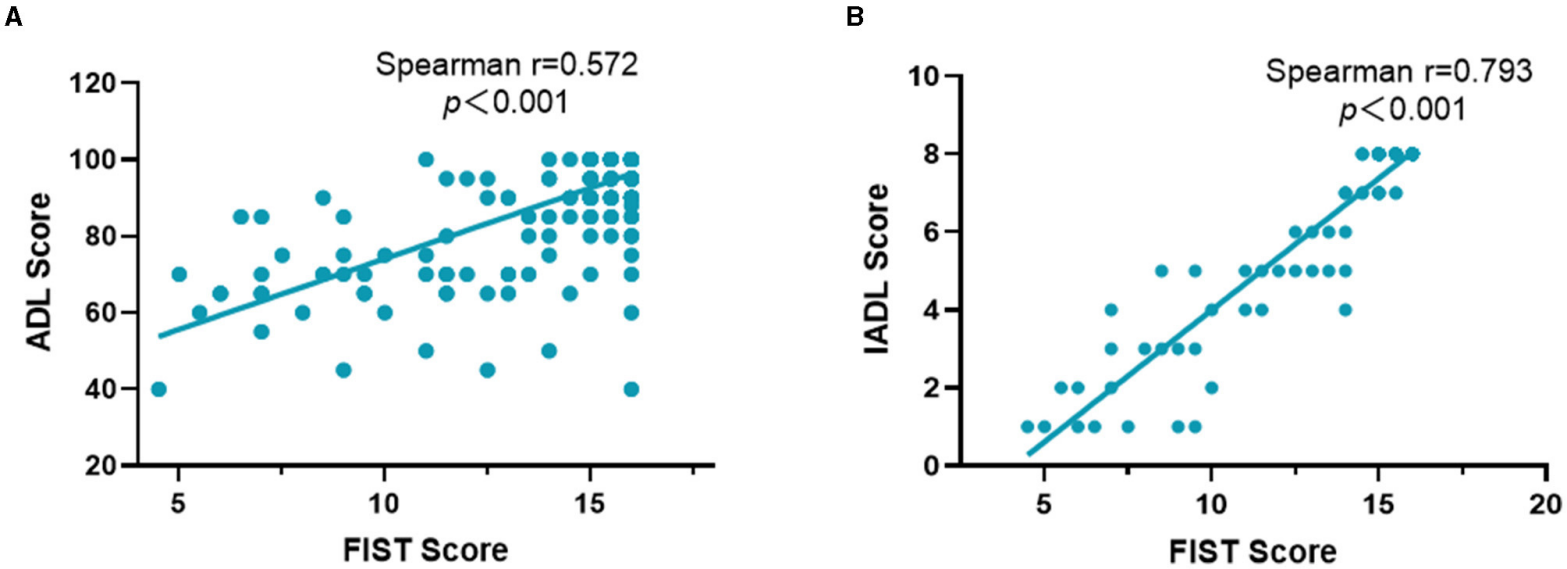
The Spearman's correlation between the FIST and ADL **(A)**, IADL **(B)**. ADL, the activities of daily living; FIST, function impairment screening tool; IADL, instrumental activities of daily living.

Criterion-related validity was assessed by Spearman's correlation between the FIST and ADL, IADL. The correlation coefficient was 0.572 (*P* < 0.001) between the FIST scores with ADL and 0.793 (*P* < 0.001) between the FIST scores and IADL (Figure 2). Based on the absolute value of Spearman's correlation coefficient, the FIST had a moderate correlation with ADL and a strong correlation with IADL.

**Figure 3:**
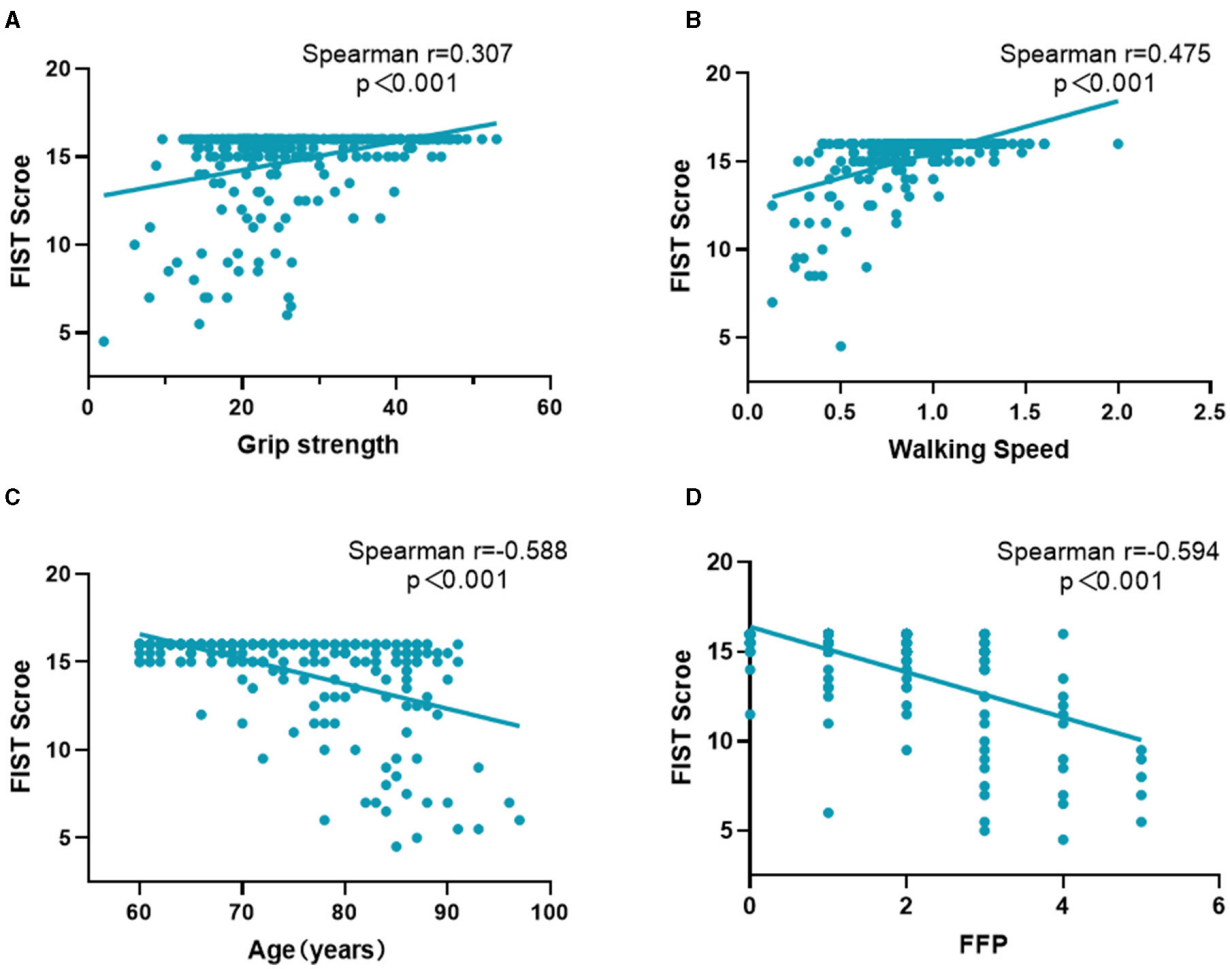
The Spearman's correlation between the FIST score and age, physical performance, physical frailty. **(A)** Describes the correlation between the FIST score and grip strength; **(B)** Describes the correlation between the FIST score and walking speed; **(C)** Describes the correlation between the FIST score and age; **(D)** Describes the correlation between the FIST score and the Fried frailty phenotype. FFP, the Fried frailty phenotype; FIST, function impairment screening tool.

Figure 3 shows the correlation between the FIST score with age, physical performance, and physical frailty. The FIST score was positively correlated with walking speed (*r* = 0.475, *P* < 0.001) and grip strength (*r* = 0.307, *P* < 0.001) and negatively correlated with age (*r* = −0.588, *P* < 0.001) and the Fried frailty phenotype (*r* = −0.594, *P* < 0.001; Figure 3).

## Discussion

To evaluate the physical functional status in Chinese older adults, we developed the FIST using the Delphi method. The present study validated the new tool in Chinese older adults and showed that FIST was a reliable instrument to assess physical function impairment.

We previously developed a scale item pool based on a literature review and formed a preliminary scale through a two-round Delphi expert consultation (18). Next, the preliminary scale was used with older participants, and the FIST scale was developed using classical statistical methods (19). The FIST scale contains 16 items that belong to three domains of functional ability (ability to perform activities of daily living, ability to engage in domestic life, and ability to engage in social activities).

The instrument's reliability is the degree of measurement accuracy, and we evaluated the reliability of the FIST in three ways: internal consistency reliability, split-half reliability, and test–retest reliability. The internal consistency reliability and split-half reliability of the FIST were excellent. The Cronbach's alpha for the FIST was 0.930, and the split-half reliability was 0.924, meeting the recommended value range 0.70–0.95 (29, 38). These results were similar to those of other functional instruments. The internal consistency for the AADL overall scale was 0.80 in older adults in Brazil (17). The Cronbach's alpha coefficient of the Frenchay Activities Index was 0.83 in non-stroke older participants, 0.78 in pre-stroke participants, and 0.87 in post-stroke participants (39). Internal consistency reliabilities for the functional status questionnaire six subscales (basic ADL, intermediate ADL, mental health, work performance, social activity, quality of interaction) ranged from 0.64 to 0.82 in 1,153 outpatients (40). The test–retest reliability of the FIST scale was 0.928, which exceeded the recommendation (>0.75) (29, 41). The retest result was an ICC of 0.928, indicating that the FIST was stable. Thus, the FIST scale shows good reliability.

Exploratory factor analysis showed that FIST has good construct validity; the factor loading values of the 16 items were all >0.40, and the contribution rate of a common factor was 60.14%. In the exploratory factor analysis, according to the scree test, we retained one factor that variance contribution rate was 60.074% that was different from the three domains of the FIST. But the load values of each item were >0.4. No item was deleted according to the statistical analysis. As our participants were older inpatients, they had worse functional status than community-dwelling older adults. The FIST should be further refined in the future and verified in different populations, especially community-dwelling older adults. Meanwhile, we also evaluated the criterion validity. There is no gold standard for functional impairment, so we analyzed the correlation coefficients between the FIST and ADL, IADL, which showed that the FIST has a moderate correlation with ADL and a strong correlation with IADL.

A previous study showed that the Groningen activity restriction scale (GARS) and the modified Reintegration to Normal Living Index were reliable and valid tools for use in older adults both in hospital and community (42). GARS contained 11 ADL items and 7 IADL items (43). Compared with GARS, the FIST scale includes not only ADL and IADL items but also items regarding social activities.

With advancing age, physical function gradually deteriorates, age was a significant predictor of functional decline (44). Our results showed that the FIST score was negatively correlated with age. Frailty is a geriatric syndrome that increases vulnerability, and it is related to a series of adverse outcomes including falls, disability, and mortality (45, 46). Our results revealed that the FIST was negatively associated with frailty. However, the function impairment was different from frailty, although some items of FIST were similar to frailty. The FIST contains not only ability in daily living but also ability in domestic life and social activities.

Both muscle mass and muscle strength decreased gradually with aging, and strength declined faster than mass (47). Muscle strength and mobility ability are important components of physical performance. Walking speed is an objective measure of physical performance. Our study showed the FIST score was positively correlated with walking speed and grip strength. Several studies have reported that walking speed was associated with risks of functional dependence and disability (48–52). Furthermore, a 6-year follow-up study found that walking speed can predict the onset of functional dependence in rural older adults (48). Another study with 4.4 years follow-up found that Japanese community-dwelling older adults with faster walking speed had decreased risks of disability (adjusted hazard ratio [HR]: 0.87, 95% CI: 0.82–0.93) (49). Furthermore, slow walking speed increased the risk of future disability in frail (adjusted HR: 4.68, 95% CI: 2.72–8.05) and prefrail older adults (adjusted HR: 3.62, 95% CI: 2.19–5.96) (50). Muscle strength is also important to maintain physical function. Greater handgrip strength was associated with decreasing onset of ADL disability and IADL disability (51, 52). Thus, the FIST may reflect physical function. The diagnosis of sarcopenia, a progressive and generalized skeletal muscle disorder, includes the assessment of muscle strength, muscle mass, and physical performance (53, 54). Although the FIST score is positively correlated with muscle strength in the present study, the functional impairment is different from sarcopenia. If muscle strength is below the reference values, sarcopenia would be suspected, and the next diagnosis procedure would include the measurement of muscle mass (55).

### Limitations

This study has several limitations. First, the participants were from a single-center general hospital in Beijing and did not provide a broad representation of all older adults in China. Further multi-center studies are warranted. Second, this is a cross-sectional study, and therefore the predictive validity of the FIST was not determined. Third, the assessment was conducted by several staff members, so measurement bias may have occurred; however, all the staff were trained using the same procedures to minimize the bias.

## Conclusion

This study provided the validity and reliability evidence of the FIST. The results showed that the FIST is a reliable and valid instrument for assessing physical functional impairment in Chinese older adults. In addition, the assessment of function impairment in the early stage of disease could reduce the adverse outcome and improve the quality of life among older adults.

## Data Availability Statement

The raw data supporting the conclusions of this article will be made available by the authors, without undue reservation.

## Ethics Statement

The studies involving human participants were reviewed and approved by Ethics Committee of Xuanwu Hospital Capital Medical University. The patients/participants provided their written informed consent to participate in this study.

## Author Contributions

YuL and LM contributed to conception and design of the study. PL, YP, YiL, and LZ organized the database. YZ performed the statistical analysis and wrote the manuscript. All authors contributed to manuscript revision, read, and approved the submitted version.

## Funding

This work was supported by National Key R&D Program of China (2018YFC2002101 and 2018YFC2002100) and Beijing Municipal Health Commission (Jing2019-2).

## Conflict of Interest

The authors declare that the research was conducted in the absence of any commercial or financial relationships that could be construed as a potential conflict of interest.

## Publisher's Note

All claims expressed in this article are solely those of the authors and do not necessarily represent those of their affiliated organizations, or those of the publisher, the editors and the reviewers. Any product that may be evaluated in this article, or claim that may be made by its manufacturer, is not guaranteed or endorsed by the publisher.
